# A Qualitative Study to Explore the Life Experiences of Older Adults in Oman

**DOI:** 10.3390/ejihpe13100150

**Published:** 2023-10-03

**Authors:** Bushra Rashid Al-Ghafri, Rawaa Abubakr Abuelgassim Eltayib, Zahir Badar Al-Ghusaini, Maram Qasim Al-Nabhani, Abdulaziz Al-Mahrezi, Yaqoub Al-Saidi, Hamed Al-Sinawi, Ahmed Mohammed Al-Harrasi, Moon Fai Chan

**Affiliations:** 1 Department of Family Medicine and Public Health, College of Medicine and Health Sciences, Sultan Qaboos University, Muscat 123, Oman; s25366@student.squ.edu.om (B.R.A.-G.); rawaaqassim@gmail.com (R.A.A.E.); maram.alnabhani95@gmail.com (M.Q.A.-N.); yaqoubs@squ.edu.om (Y.A.-S.); 2 Department of Arabic Language and Literature, College of Arts and Social Sciences, Sultan Qaboos University, Muscat 123, Oman; zahir@squ.edu.om; 3 Director General, Sultan Qaboos University Hospital, Muscat 123, Oman; abdulaziz@squ.edu.om; 4 Department of Behavioral Medicine, Sultan Qaboos University Hospital, Muscat 123, Oman; senawi@squ.edu.om (H.A.-S.); drharrasi@squ.edu.om (A.M.A.-H.)

**Keywords:** reminisce, older adults, qualitative, narrative, life review, Oman

## Abstract

Background: Reminiscence studies and life reviews have a number of proven advantages. Future generations gain by learning from elders’ life experiences, as do older adults themselves who share their memories. Despite Oman’s sizable geriatric population, research on older individuals’ life experiences is scarce. Therefore, this study aimed to explore the life experiences of older Omani individuals across their many life stages, from childhood to the present. Methods: This was a qualitative study design. Convenience sampling was employed and conducted from December 2021 to October 2022. A total of 13 Omani older adults (9 females and 4 males), with an average age of 68 years, were recruited for this study (response rate = 34%). Socio-demographic and life review information was gathered according to a set of semi-structured guiding questions. The responses were then captured on audio recordings, which underwent transcription and translation. Thematic analysis techniques were applied to the extracted data. Results: Three main themes were evident in this study’s findings: childhood memories, friendships, and relationships, as well as the elders’ past. Additionally, older adults passed on a number of gems of wisdom to be shared with the younger generations. Conclusions: This study aided in revealing the resiliency, social connections, and life reflections of Omani older adults. These themes can guide the creation of age-inclusive laws, social support initiatives, and healthcare services specifically designed to satisfy the special requirements and ambitions of the elderly population. Based on these themes, this study recommended that the local community or society build a more sympathetic and compassionate atmosphere that honors and respects the accomplishments of this essential group by recognizing and comprehending the complex experiences of older adults. In addition, future studies could explore particular aspects of these older experiences and pinpoint solutions to improve their quality of life and wellbeing.

## 1. Introduction

Elders constitute an important component of our society but are often underrepresented in research. Worldwide, the elderly population has been increasing. Individuals aged 65 and above represent around 9.8% of the global population, a significant increase from the 6% it used to be in the 1990s [1]. Hence, this points towards older adults being a significant demographic to which much attention should be paid. Everybody eventually goes through the natural process of aging. Growing older involves going through many life experiences, both pleasant and terrible. These encounters will produce a wealth of information and experience that must be shared with the world.

Several qualitative studies worldwide were undertaken to explore these experiences that older adults have gone through. For example, a study by Rokach and Berman [2] interviewed 132 Canadian seniors and extrapolated several crucial insights on life and words of wisdom for future generations. Hall [3] conducted qualitative interviews with British and Japanese seniors in retirement migration to assess the challenges people face when receiving care services due to language, culture, location, and cost. Moreover, Stuart [4] undertook a life story study of senior people, and the results helped healthcare professionals by knowing about life transition experiences. Inquiring about older adults’ memories and documenting their responses is called life review or reminiscing in the literature. However, sometimes, life review and reminiscing are used interchangeably by researchers. In actuality, reminiscing involves a more passive and spontaneous recall of the past, while a life review typically revolves around a more structured and deliberate procedure. The latter process usually either has a therapeutic or an educational intention to its initiation [5]. Erikson [6] developed a theoretical framework for the ego development of an individual. In general, Erikson proposed that every individual has eight psychosocial life stages that correspond to four main physical developmental stages, namely childhood (trust versus mistrust, autonomy versus shame and doubt, and initiative versus guilt), adolescence (industry versus inferiority and identity versus role confusion), adulthood (intimacy versus isolation and generativity versus self-stagnation), and old age (ego integrity versus despair). Butler further developed Erickson’s work by proposing that ego integrity can be attained through recalling one’s past in an analytical and evaluative way, indicating the important function of a life review in the successful adaptation of older adults [7]. Butler further introduced how a life review could help re-examine positive and negative life events and critically evaluate the individual’s past. Studying older adults’ life experiences benefits future generations and older adults who reminisce about their lives. One documented benefit of encouraging older adults to recall their memories is that it significantly reduces depressive symptoms in those participating [8,9,10]. Even short-term life reviews on cancer patients have demonstrated usefulness in significantly enhancing their spiritual wellness and alleviating their suffering [11,12]. Additionally, it has been noted that facilitating older people to reflect on their lives may encourage them to accept their pasts more [12,13,14] and help them prepare for death with noticeably less fear of the inevitable outcome [15]. According to a study by Hommelhoff et al. [16], retiree wellbeing is not only influenced by outside influences, but also by what seniors reminisce on and recollect.

In Oman, the elderly population has become an important demographic to which much attention must be paid. It has been reported by the Statistical, Economic and Social Research and Training Centre for Islamic Countries (SESRIC) [17] that the senior population has grown from around 18,232 in 1960 to 126,243 in 2022, and that almost 2.7% of the population in Oman was aged 60 or above in 2022. Furthermore, it has been noted that 3.6% of females fall between the ages of 65 and above, while 2.1% of males in the population are 65 and older [17]. A study in Oman reported an increasing demand for older adults who needed more primary healthcare services [7]. These services included physical fitness-related care, psychological health care, and community support, positively impacting their quality of life. Another local study explored the changes in the psychological issues of older adults in the post-COVID-19 pandemic [10]. This study revealed a positive impact of the preventive measures on the quality of life and reduced depressive symptoms. Despite the noteworthy proportion of older adults in Oman, studies are scarce investigating older adults’ life experiences. Thus, this study aimed to explore the life experiences of older Omani adults during their different life stages.

## 2. Materials and Methods

### 2.1. Study Participants

The research comprised a group of 13 senior citizens from Oman, consisting of 9 females and 4 males, with an average age of 68 (ranging from 60 to 79) years. These individuals volunteered to participate in this study and were selected from the medical clinic at Sultan Qaboos University in Muscat, Oman. In order to be eligible, participants had to be of Omani nationality, proficient in speaking and understanding Arabic, and willing to provide informed consent. This study focused on individuals aged 60 and above, aligning with the definition of older adults in Oman. Those with conditions like Parkinson’s disease, dementia, Alzheimer’s, or significant mental disorders were not included. Initially, 50 older adults were approached for participation. However, after applying the criteria, 33 participants were excluded, as 16 did not meet the requirements and 17 opted not to take part, resulting in a response rate of 34%. Furthermore, 4 participants opted to withdraw from the study, making their data discarded as requested.

### 2.2. Data Collection

Convenience sampling was employed and conducted from December 2021 to October 2022. A researcher (BRAG) was stationed in the community center at Sultan Qaboos University and invited potential subjects to join the study while waiting for their medical appointment. This researcher has a health-related MSc qualification and is conducting her PhD for this study. This researcher has more than 5 years of working experience in the healthcare organization in Oman. Before conducting the interview, she received a 2 days training course on interviewing older adults trained by her main supervisor (MFC), who has previous experiences conducting similar studies in other countries. After training the researcher, one older adult was conducted to have a pilot study to ensure that the procedures followed the protocol, and no discrepancy was found in this pilot study. Prior to the invitation, the researcher did not know the participant’s background information. She introduced herself with a name batch which indicated that she is a researcher of the institution. Each eligible participant was informed about the study, and before beginning the interviews, which took place once a week for four weeks, a signed agreement was obtained. Participants were told they could withdraw from the study and that their data would be destroyed immediately to ensure their privacy.

### 2.3. Ethical Considerations

Ethical approval was obtained from the medical research ethics committee of Sultan Qaboos University (MREC #2028). Before obtaining their signed informed permission, each participant was able to ask questions about this study. An individual number was used to ensure privacy and secure participant identities.

### 2.4. Procedures

#### 2.4.1. Socio-Demographic Variables

In the first survey, socio-demographic variables, such as gender, age, marital status, occupation, education level, living status, having medication, kind of chronic diseases, frequency of meeting relatives and friends, and physical activity per week, were obtained.

#### 2.4.2. Life Review Questions

This study was based on the conceptual framework of Erikson [6] and Butler [7], which had four phases: childhood, adolescence, adulthood, and old age. The participants’ homes were the venues for the meetings. The participants had four meetings (one for each phase) of around 1 h each. The semi-structured questions used in this study were adapted from a previous study conducted by Chan et al. [18] and also one of the researchers of this study (MFC). He developed several questions on a life story review in a group of older adults in Singapore based on the theory developed by Erikson [6] and Butler [7]. These questions covered all phases. The authors (ZBAG and BRAG) reviewed the questions for each stage to ensure that the questions of each phase met the local context and were discussed with team members. Once the team agreed, these questions were translated into Arabic from English, and another researcher back-translated them into English (RAAE). To ensure that the meaning between both versions was consistent, it was confirmed by another researcher (MFC). A pilot study was conducted to ensure that the procedures followed the protocol, as stated in Section 2.2. Participants fully understood the meaning of these questions at each meeting.

Samples of the questions on each phase (meeting) included the following: First meeting—childhood: “Do you have warm feelings about the childhood home that you remember the most?” and “What were some of the best memories of special events you celebrated?”. Second meeting—adolescence: “What was your favorite activity with friends together?” and “Was there any teacher who made a special impression on you?”. Third meeting—adulthood: “How would you describe the relationship with your spouse?”, “What memories would you like to share with your children or grandchildren that you never have before?”, “How do you see your role as a housewife? (female only), and “How important was your career in your life?”. Fourth meeting—old age: “What is the meaning of life?”, “What is the most important thing you have learned in your life?”, and “Is there anything that you would like to change in the past?”. 

#### 2.4.3. Procedure to Record the Interview

The interviews with the participants were face-to-face and took place at their home and were recorded using audio tape without video capture. Only the researcher and the participant were involved in the data collection without other people involved. In addition, field notes and memos were written down for each interview. The total time spent was around 4 h within 4 meetings. The recorded information was transcribed into words and translated from Arabic into English.

### 2.5. Data Analysis

The principal qualitative data technique used in this study was thematic analysis [19], which has been defined as a method “for identifying, analyzing, and reporting patterns (themes) within data” [19]. It organizes and describes the data collection in (rich) detail at a high level. “Frequently, it goes further than this and interprets various aspects of the research topic” [19]. To analyze the data, the following procedures were taken: becoming familiar with the data, creating initial codes, arranging codes, looking for themes, reviewing, and defining/naming themes/subthemes. One researcher (MQAN) manually coded each transcript line-by-line, including the memo note, to complement the coding procedures. The codes, data interpretations, and data saturations were discussed with another researcher (BRAG). With the thirteen participants, data saturation was reached, and data gathering was halted. All transcript comparisons were made throughout the coding process to identify themes. Similar meanings from the codes were grouped into categories and were named into meaningful subthemes/themes [19,20]. Microsoft Excel and Microsoft Word 2013 were used for data analysis. 

### 2.6. Rigor

The research team examined the data in five areas to improve the validity and reliability of the results: truth value, applicability, consistency, validity, and reliability [20]. They verified the truth value of the audio files from the interview by checking for missing or distorted parts. Data was collected and analyzed to ensure high applicability until interviews stopped generating new information that had not yet been discovered in prior interview data analyses [19]. This was the point at which data saturation was determined to have occurred. A researcher (ZBAG) who trained a research assistant to do the transcription was cross-checked by another researcher (BRAG) for accuracy. They met with the participants to check whether the transcripts corresponded to the statements for consistency [21]. Another researcher (RAAE), a medical doctor with bilingual translation experience, helped with the translation and editing, considering the linguistic differences between Arabic and English. Furthermore, for triangulation, researchers (RAAE, BRAG, and MQAN) discussed the interview and data analysis methods several times to ensure consistency and rigor for the study [22]. They also shared feedback and ideas with the participants to ensure that the results were valid and reliable and captured the core and meaning of the study’s theme [19]. After finishing the analysis of the results, a bilingual translator (RAAE) translated them, and another researcher (BRAG), proficient in both languages, conducted the secondary editing. The researchers then employed back translation to detect any discrepancies between Arabic and English. If there was any disagreement, they discussed it until they reached a consensus.

## 3. Results

### 3.1. Descriptive Characteristics

In this research, 13 elderly participants were interviewed for their life stories. A summary of the descriptive statistics of the participants has been presented in Table 1. Eight out of thirteen participants were between sixty and sixty nine years of age, whereas the remaining five older adults were aged from seventy to seventy nine. Additionally, the majority of the interviewed elderly were females (69.23%), while only 30.77% were males. Seven interviewed older adults were married, five were widowed, and only one was divorced. All participating older adults in this study lived with their families except for one elderly adult who lived alone. Regarding the participants’ educational background, almost three-fifths were either illiterate or only had a primary school education. Moreover, approximately 23% of the interviewed elderly have received a middle school-level education, whereas only two elderly participants either had a high school or higher education. Concerning their occupation, six of them were retired (46.15%), another six were currently working as housekeepers (46.15%), and only one of the interviewed older adults was unemployed (7.7%). Furthermore, seven interviewed participants reported having a monthly income (53.85%), whereas six reported being without income (46.15%). All participants in this study had chronic diseases. Most of the older adults reported having diabetes (69.23%), while 53.85% reported having hypertension, and only 38.46% suffered from high cholesterol.

### 3.2. Thematic Analysis Results

Next, the interview results were disseminated into three themes, as illustrated in Table 2, with the major points highlighted under each theme.

#### 3.2.1. The First Theme: Childhood Memories

When interviewed about their childhood memories and experiences, many interesting things were apparent regarding the usual childhood experience of the Omani elderly.

A. Childhood homes: When the elderly participants were asked if they remembered their childhood homes and whether they missed them or not, there were some contrasting opinions. Some of the interviewed older adults mentioned that they missed their childhood homes, examples of which include the following:

“*Yes, I miss it. It has my memories*”

[F2, line 44].

“*Who does not miss their childhood, any person when they are alone, they relive their memories*.”

[M3, line 32].

Some elderly participants reported that they miss their homes but are unable to visit those houses:

“*Yeah, but they demolished it*”

[F4, line 22].

“*Yes, I miss it and long for it, but I cannot return to it because of my husband’s illness*”

[F3, lines 32 and 34].

“*Actually, after I built my new house, I cannot go back to live in that house because no one lives there anymore. My father passed away, and my mother lives at my sister’s house.*”

[M1, line 36].

Interestingly, a few older adults revealed that they did not miss their childhood homes, examples of which include the following statements:

“*A house made of cement. I do not really miss it because it is a small house and so on. I only miss the gatherings in it.*”

[F8, line 21].

“*No, I do not miss it. Do you know why? You know, in the past, it was a mud house, and our area was cold at the green mountain, a very cold area*”

[F9, lines 44 and 46].

B. Favorite childhood memories: When seniors were questioned about their most cherished childhood memory, most mentioned the Eid holidays, examples of which include the following statements:

“*Holidays (Al-Fitr and Al-Adha), and Birthdays*”

[F3, line 41].

“*As for the holidays, we perform the Eid prayer and then greet people at the mosque, after which we greet family and relatives. The rituals of Eid al-Adha start on the ninth day, in which we slaughter livestock, and on the morning of Eid, we cook the traditional meal (Alarsaih), and on the second day of Eid, we slaughter the livestock and cook skewers and grilled meat*.”

[M2, lines 44–47].

C. Working history: Most interviewed females reported that they did not hold any job positions during their early life stages. Most of them associated that with the typical female role in the earlier generations, examples of which include the following statements:

“*No, there were no jobs before, only household work*”

[F4, line 15].

“*We used to do household work, watering, and chopping firewood*”

[F2, line 26].

“*I did not work. I did household work*”

[F7, line 12].

However, not all females are housewives, and one female reported the most extensive working experience:

“*First, I worked at a bank. Then I worked as a librarian in Thumrait, then at the Ministry of Health as a librarian, then at the Royal Hospital*.”

[F8, lines 14–15].

In contrast, when asked about their employment history, the male participants reported drastically different experiences. All of the males that were interviewed had some working history in a variety of occupations, examples of which include the following statements:

“*I used to work as a correspondent at Oman Development Bank*”

[M1, line 20].

“*My job was a civil guard at the university*”

[M2, line 22].

“*Director General in the Ministry of Education*”

[M3, line 13].

“*I worked at Oman Air in the equipment department, specializing in aviation equipment*.”

[M4, line 8].

#### 3.2.2. The Second Theme: Friendships and Relationships

The goal of this segment was to elicit information from the elderly Omani volunteers on the friendships and relationships that they nurtured throughout their lives.

A. Childhood friendships: The elders were questioned whether they remembered their childhood friends. Some of the seniors remarked that they still had good recollections of their friendships from their childhood days. Although some people have been able to keep up with their friends thanks to recent technological advancements, others have not, examples of which include the following statements:

“*Yes, I have childhood friends, but now we do not communicate with each other because there were no phones before and also because we were busy and separated in different provinces as a result of marriage*.”

[F7, lines 33–34].

“*My old friends, some dead and some alive, and others moved far away, I do not know where. I still communicate with some of them until now through WhatsApp as these applications bring everyone closer.*”

[M4, lines 36–37].

On the other hand, several elderly individuals stated that they do not remember their friends from their childhood period:

“*Those from my childhood days, I do not remember because most of them have passed away*”

[F2, line 68].

“*Childhood friends, I do not remember them. As for friends from recent years, yes, we visit from time to time*”

[F3, line 46].

B. Remembrance of childhood teachers and the importance of education: Several elderly seniors acknowledged the importance of education, particularly when overcoming some hardships to obtain it. In addition, many other seniors spoke highly of their prior schooling and how it benefited them both personally and professionally:

“*Yes, it is important. It is true that it was tiring and there was no electricity, and we had to bring water from the well. But we endured and armed ourselves with knowledge and obtained a degree*”

[F1, line 87].

“*I learned how to raise my children, right from wrong, how to talk, and how to take care of my health and deal with people. There is a difference between people who studied and those who did not study or have not completed their studies.*”

[F9, lines 107–108].

“*Thanks to God, we studied and completed high school. We were not employed or went to college. I got married and returned to Oman. Thanks to God, my high school diploma helped me find a job, so I worked in banks, ministries, and companies*.”

[F8, lines 54–55].

“*Its importance is me reaching where I am now. If it were not for education would not have reached it*.”

[M3, line 59].

Despite some elders mentioning that they have not completed their formal education, they recognize its importance. They are glad it aided them in their religious journey as it assisted them to be able to read and memorize their holy book “*Quran*”, as two seniors mentioned:

“*Very useful I read, write, recites the Qur’an, and manage my affairs*”

[M4, line 56].

“*Yes, those years were of great importance in my life. It is true that I stopped after middle school, but that period is enough to teach us a lot. We learned the Qur’an and memorized and understood many aspects of life. Knowledge enlightens the person*”

[M2, lines 62–63].

“*I remember teacher Karima who used to teach me Arabic*”

[F3, line 59].

“*Yes, in Muscat, teacher Adel Al-Qadi. He was a principal at Al-Saidia School. I also remember Professor Tawfiq. May God rest his soul in peace, and Professor Ramsey, my teacher at Al Saidia School in Muscat*.”

[M3, lines 51–52].

C. Marriage difficulties: When the participants were questioned about whether they encountered any marriage difficulties in their lives, interestingly, most cited that they faced several difficulties with their spouses. Several participants indicated that the main precursor to their marital problems was a shortage of money, examples of which include the following statements:

“*Yes, indeed, we have been through hardships. In the past, I did not have enough money to build a house for my family, and my wife wanted to move to a private house….*”

[M2, lines 68–70].

“*Yes, at the beginning of our marriage, we were young, and he was not even employed after we had our first child.*”

[F5, line 50].

“*Yes, sure, he did not have anything. In Abu Dhabi salary was only 40 riyals. His mother and 2 brothers were living with us. Life was difficult before we came back from Zanzibar. My mother used to work as a baker, cooking Mandazi and selling it. My husband used to bring a truck from Dubai to Oman. It has vegetables, and that was his job, and we used to work and do everything*.”

[F4, lines 65–68].

Additionally, other elderly participants cited that their marital issues were related to their children:

“*We fought a little bit about the issue of children. In the beginning, God blessed me with three daughters while he wanted a son*”

[F3, lines 70–71].

“*Oh yes, we went through difficult times… and after God chose my husband, the circumstances became much more difficult for me because my responsibilities increased and my children were young*.”

[F6, lines 63–64].

D. Significance of the seniors in the lives of those around them: The majority of the elderly participants were aware of their importance in other people’s lives, as one female senior mentioned:

“*Thank God it is good, and when I am not at home for a couple of days, they say that the house is not great without you, and my husband does not know how to manage things without me*.”

[F1, line 99].

“*I was kind to my husband, and after his death, I raised my children by myself, and I was patient throughout difficult circumstances*.”

[F2, line 96].

“*I treated them well. They say that I sacrifice for the sake of others. I greeted everyone, and everyone asked about me*.”

[F3, line 80].

E. Significance of older adults’ role as parents: All of the older adults who were interviewed acknowledged the value of their parental responsibilities and the joy and satisfaction that came with it, examples of which include the following statements:

“*I love being with my children, having them around me, and our gatherings together*”

[F3, line 84].

“*Yes, I was excited about this. They are the light of the house; children are a blessing from the Lord. Educating them, trying to get them residential lands, and marrying them off. Ensuring their stability is the best responsibility for me*.”

[M1, lines 72 and 76].

“T*hey made me happy and filled my life because I did not have anyone*…”

[F4, line 87].

#### 3.2.3. The Third Theme: The Older Adults’ Past

This segment of the study was concerned with prompting the elderly Omani individuals to reflect on their pasts, including painful experiences they had, whether they were happy with their lives thus far, whether they had any regrets, and what the most important lesson in life was that they wanted to impart to future generations.

A. Acceptance of the past: Older adults seemed to accept the past events that occurred during their lives. Although some people’s pasts were filled with misery, they are currently at peace with them and content with their lives thus far, examples of which include the following statements:

“*No, despite the difficulty of life, and we when to collect firewood, irrigate, and reside in tents, the past was good, and I do not want to change anything from it*.”

[F2, line 131].

“*No, I do not wish for it to return, life in the past was poverty and misery, and no one wishes for it to return. As for now, we live in prosperity and abundant provision, thanks God*.”

[M2, line 94].

However, some seniors wished that they could return to instill more wisdom in themselves and their children or spend more time with a loved one who had passed away. Some highlight quotes from the interviewed participants include:

“*To learn and teach my children to save up”*

[F5, line 76].

*“If I were to go back in time, I would wish the mother of my children would stay by my side, but it is God’s command*.”

[M1, line 92].

“*I wish time would come back*”

[M4, line 90].

“*A long time ago, we did not wear headscarves, and we worked with men. When I remember this, I think about why we did this, and I regret it. May our Lord forgive us*”

[F8, lines 106–107].

B. The saddest memory: When older adults were asked to name their saddest remembered events, most of them described a time when they had lost a loved one. The death of a parent, child, or partner seemed to have the greatest impact on the seniors interviewed in this study, examples of which include the following statements:

“*The death of my father and mother. They are irreplaceable as they had imprints on my upbringing*.”

[M3, line 98].

“*My father’s death. I was eight months pregnant. He went on vacation while I was pregnant. He called me, and I told him that we missed him. He jokingly said that he would come back if I had a girl. Three days later, the news came that my father had passed away*.”

[F8, lines 111–112].

“*When my children, Al-Khattab, died in an accident in 2008, then Qusay*.”

[F4, line 115].

“*Sadness is inevitable in life, such as the death of relatives*.”

[M1, line 98].

C. Key lessons from their life: The interviewed elders conveyed many lessons from their experiences. One of the imparted pearls of wisdom recommended was to respect other people, as one female senior mentioned:

“*I learned to respect people, respect my husband, love my children, and do everything that pleases God*.”

[F1, line 155].

The second pearl of wisdom imparted upon us, which older adults seemed to agree upon, was the virtue of patience:

“*Live your life with joy and be patient in difficult circumstances. Do not despair, as patience is the key to relief*”

[F3, line 95].

“*I learned from this life patience through hardships, respect for others, and self-confidence*.”

[M2, line 98].

“*Life has taught me patience, humility, and contentment. And that I do not look at others, but I say may God bless me like them*.”

[F6, line 85].

“*We lived a difficult life, which taught us patience and strives*”

[M4, line 96].

## 4. Discussion

### 4.1. Discussion on Themes

In general, this study sought to interview older Omani adults to learn about their lives and experiences to glean some important wisdom and, perhaps inadvertently, help older adults feel more content with their accomplishments and lives. Several key themes that have relevance for comprehending the experiences of the elderly were noted during the interview sessions.

The first theme that emerged was concerning childhood memories. The seniors in this study seemed to have a turbulent relationship with their childhood homes. It is clear from the interview responses that elderly individuals who had happier childhoods tended to associate these fond recollections with their childhood residences. The contrary was also true; individuals who had somewhat of a rough or turbulent upbringing saw their childhood homes as nothing but pieces of mud or concrete and did not miss them. The literature also highlights this theory, informing us that childhood homes are a stable setting for comfort, safety, and family [21,23]. This was further emphasized when older adults were asked to cite their most cherished childhood memories. Almost all of them recounted when their families were together, like at Eid or birthday gatherings, where they had pure, loving, and supportive interactions. Therefore, it is imperative to communicate this advice to newly formed families, urging them to put forth every effort to give their kids the happiest, safest childhood experience imaginable. Intriguingly, the seniors revealed a phenomenon throughout their recollections that accurately captured the experience of older generations when society was less accepting of certain viewpoints [2]. It was highlighted through the recounts that most female older adults were not expected to hold any working positions. They were merely expected to follow the societal conventions of their time, which meant dedicating their time to caregiving and performing duties in or around their homes [22]. However, the opposite was true when it came to the senior males in this study. All the men who participated in the interviews had previous employment histories. It is believed that cultural origins are one of the primary causes of this phenomenon. Western women have made great progress in their daily lives since the 1800s, moving from simple domestic responsibilities to management and leadership positions. However, this is still a somewhat recent development in the Middle East [24,25].

The second theme in this study was related to the relationships and friendships of older adults throughout their lives. Social networks significantly influenced the senior participants’ lives [22]. When asked about one of their earliest memories of friendships, older adults responded with mixed responses, specifically regarding whether they can still remember their early childhood friends [3]. Some interviewed individuals could clearly remember their childhood friends and even overcame technological and communication difficulties to keep their old friendships going. This indicates the importance that older individuals place on maintaining relationships with their childhood friends and their commitment to maintaining the social networks created during childhood [26,27]. This point was further emphasized when the participants noted how significantly their teachers impacted their educational journey throughout childhood and adolescence. Many of our participants were able to vividly and fondly recall the teachers who taught them in the early days.

Additionally, practically all of the interviewed seniors acknowledged the value of education, given that without it, most of them would not now be in such good positions, both personally and professionally. These findings were similar to another study in America [22]. Interestingly, older adults cited that they experienced marriage difficulties. Some of the problems mentioned by older adults were due to circumstances beyond their control, such as financial difficulties or sickness. At the same time, other issues might have been resolved with improved understanding, empathy, and communication between the couples in question [26]. In the modern era, social media and mainstream media frequently present an idealized view of marriage, fostering the impression that everything should go smoothly. However, the elders’ tales demonstrate no relationship without challenges [28].

Furthermore, older people offer a realistic perspective on the difficulties of marriage by sharing stories of their hardships and how they overcame them. This realism can encourage younger generations to approach relationships with more empathy, patience, and understanding [26]. Thankfully, many of the interviewed elderly were aware of their importance in the lives of those around them. However, another study indicated that older people struggled with feelings of loneliness and unimportance in the lives of others around them [28]. Knowing your worth in other people’s life is important at any age, but it is particularly vital for older adults. According to an Iranian study that explored retirees’ life stories, one of the factors contributing to the wellbeing of older people is their realization of their value in the lives of those surrounding them [20]. The sense of purpose in life was further reinforced when nearly all older adults stated that they understood the worth of their parental obligations and the joy and satisfaction that came with it. This was consistent with the results of an investigation conducted by Sharma and Bluck [29], which explored the sense of purpose in older adults, how they developed it, and what aided them in maintaining it. This study underlined that one of the elements that helped seniors achieve their life goals was cultivating relationships. Overall, this theme highlighted the significance of preserving deep connections with family and friends and cherishing those relationships. These findings were similar to other studies [22]. Preserving interpersonal and familial connections is especially crucial for senior citizens, as numerous studies have shown that seniors who experience emotions of loneliness, a lack of close companionship, and sentiments of unimportance frequently have more depressive symptoms as they age and believe life has no purpose [27,28,30].

The third theme concerned Omani seniors’ past. Older adults appeared to have come to terms with their past experiences. Although several participants had unpleasant pasts, they are now at peace with them and happy with their present circumstances. Those who were not accepting of their pasts were due to them wishing they could go back and deal with certain situations in a better, more wisdom-filled way or spend more time with loved ones who passed away. Learning to deal with regrets can be seen as an experience that benefits the individual as it may improve one’s wellbeing [31]. In life, one must learn to cope with pain and loss. Most older adults spoke about how losing a family member or a loved one was the saddest and most trying time of their life. However, one of the key traits exhibited by older adults in this study was their admirable perseverance in the face of difficulties in life. This was similar to a finding from another study by Lind et al. [32] that showed how resilient older people are when faced with challenges, especially during times of crises, like the COVID-19 pandemic.

Last but not least, the elders who took part were asked to identify some important lessons that their experiences have helped them learn and that they hope to pass on to future generations. There were two main pearls of wisdom that the seniors seemed to agree upon: patience and being respectful of other people. These findings were similar to other studies [2,4,28]. Hardships are common in life; thus, one should not give up in the face of them. Instead, people should be patient, persevere through them, and attempt to overcome them.

### 4.2. Strengths and Limitations

One of the main strengths of this study is that, to our knowledge, this is the first qualitative study that focused on older adults’ life stories conducted in Oman and is therefore considered a valuable starting point for future research to focus more on the lived experiences of older Omani adults and how to improve their quality of life. Another strength of this study was that it utilized the value of in-person interviews, which has been theorized to result in a greater connection between the participants and the interviewer. On the other hand, there exist some theorized limitations of the study. Although strategies were employed by the researchers of this study to reduce their prejudices, like in all qualitative studies, coding may have been influenced by personal biases.

### 4.3. Recommendations

This study recommended that the local community or society build a more sympathetic and compassionate atmosphere that honors and respects the accomplishments of this essential group by recognizing and comprehending the complex experiences of older adults. Based on these findings, future studies could explore particular aspects of these older experiences and pinpoint solutions to improve their quality of life and wellbeing.

## 5. Conclusions

In conclusion, this study reveals Omani older adults’ resiliency, social connections, and life reflections. This study offers a holistic understanding of their experiences. The themes found in this study can guide the creation of age-inclusive laws, social support initiatives, and healthcare services specifically designed to satisfy the special requirements and ambitions of the elderly population.

## Figures and Tables

**Table 1:** Descriptive statistics of the study participants.

Measure		*n*	%
Age	60–69	8	61.54
	70–79	5	38.46
Gender	Female	9	69.23
	Male	4	30.77
Marital status	Married	7	53.85
	Widowed	5	38.46
	Divorced	1	7.69
Live with	Live alone	1	7.69
	Live with family	12	92.31
Education	Illiterate	4	30.77
	Primary	4	30.77
	Middle school	3	23.08
	High school	1	7.69
	Higher education	1	7.69
Occupation	Retired	6	46.15
	Housekeeping	6	46.15
	Unemployed	1	7.7
Income	Without	6	46.15
	With	7	53.85
Medical problem	Hypertension	7	53.85
	Diabetes	9	69.23
	Cholesterol	5	38.46

**Table 2:** Main themes, subthemes, and representative quotations of perceptions.

Main Themes and Subthemes	Representative Quotations
**Childhood memories**	
A. Childhood homes	“*Yes, I miss it. It has my memories*” [F2, line 44]. “*Yeah, but they demolished it*” [F4, line 22]. “*A house made of cement. I do not really miss it because it is a small house and so on. I only miss the gatherings in it*” [F8, line 21].
B. Favorite childhood memories	“*Holidays (Al-Fitr and Al-Adha), and Birthdays*” [F3, line 41]. “*As for the holidays, we perform the Eid prayer and then greet people at the mosque, after which we greet family and relatives. The rituals of Eid al-Adha start on the ninth day, in which we slaughter livestock, and on the morning of Eid, we cook the traditional meal (Alarsaih), and on the second day of Eid, we slaughter the livestock and cook skewers and grilled meat*” [M2, lines 44–47].
C. Working history	“*No, there were no jobs before, only household work*” [F4, line 15]. “*I did not work. I did household work*” [F7, line 12]. “*I used to work as a correspondent at Oman Development Bank*” [M1, line 20]. “*My job was a civil guard at the university*” [M2, line 22]. “*Director General in the Ministry of Education*” [M3, line 13].
**Friendships and relationships**	
A. Childhood friendships	“*My old friends, some dead and some are alive, and others moved far away, I do not know where. I still communicate with some of them until now through WhatsApp as these applications bring everyone closer*” [M4, lines 36–37]. “*Those from my childhood days, I do not remember because most of them have passed away*” [F2, line 68].
B. Remembrance of childhood teachers and the importance of education	“*I remember teacher Karima who used to teach me Arabic*” [F3, line 59]. “*Yes, it is important. It is true that it was tiring and there was no electricity, and we had to bring water from the well. But we endured and armed ourselves with knowledge and obtained a degree*” [F1, line 87]. “*I learned how to raise my children, right from wrong, how to talk, and how to take care of my health and deal with people. There is a difference between people who studied and those who did not study or have not completed their studies*” [F9, lines 107–108]. “*Very useful I read, write, recite the Qur’an, and manage my affairs*” [M4, line 56].
C. Marriage difficulties	“*Yes, indeed, we have been through hardships. In the past, I did not have enough money to build a house for my family, and my wife wanted to move to a privet house…*” [M2, lines 68–70]. “*Yes, at the beginning of our marriage, we were young, and he was not even employed after we had our first child*” [F5, line 50]. “*We fought a little bit about the issue of children. In the beginning, God blessed me with three daughters while he wanted a son*” [F3, lines 70–71].
D. Significance of the seniors in the lives of those around them	“*Thank God it is good, and when I am not at home for a couple of days, they say that the house is not great without you, and my husband does not know how to manage things without me*” [F1, line 99]. “*I treated them well. They say that I sacrifice for the sake of others. I greeted everyone, and everyone asked about me*” [F3, line 80].
E. Significance of older adults’ role as parents	“*I love being with my children, having them around me, and our gatherings together*” [F3, line 84]. “*Yes, I was excited about this. They are the light of the house; children are a blessing from the Lord. Educating them, trying to get them residential lands, and marrying them off. Ensuring their stability is the best responsibility for me*.” [M1, lines 72 and 76]. “*They made me happy and filled my life because I did not have anyone…*” [F4, line 87].
**The older adults’ past**	
A. Acceptance of the past	“*No, despite the difficulty of life, and we when to collect firewood, irrigate, and reside in tents, the past was good, and I do not want to change anything from it.*” [F2, line 131]. “*No, I do not wish for it to return, life in the past was poverty and misery, and no one wishes for it to return. As for now, we live in prosperity and abundant provision, thanks God.*” [M2, line 94]. “*I wish time would come back*” [M4, line 90]. “*If I were to go back in time, I would wish the mother of my children would stay by my side, but it is God’s command.*” [M1, line 92].
B. The saddest memory	“*The death of my father and mother. They are irreplaceable as they had imprints on my up-bringing.*” [M3, line 98]. “*When my children, Al-Khattab, died in an accident in 2008, then Qusay.*” [F4, line 115]. “*Sadness is inevitable in life, such as the death of relatives.*” [M1, line 98].
C. Key lessons from their life	“*I learned to respect people, respect my husband, love my children, and do everything that pleases God.*” [F1, line 155]. “*Live your life with joy and be patient in difficult circumstances. Do not despair, as patience is the key to relief*” [F3, line 95]. “*I learned from this life patience through hardships, respect for others, and self-confidence.*” [M2, line 98].

## Data Availability

The dataset used in this research can be made available with a reasonable request from the corresponding author.
